# Alveolar Abscess

**Published:** 1871-11

**Authors:** C. Stoddard Smith


AMERICAN JOURNAL

OF

DENTAL SCIENCE.

Vol. V. THIRD SERIES--NOVEMBER, 1871. No. 7.

ARTICLE I.

### *Alveolar Abscess.*

BY C. STODDARD SMITH, D. D. S.

Read before the Illinois State Dental Society.

Until within a period commencing but a few years since, the subject of alveolar abscess was, so far as the literature of the profession was concerned, very much neglected; in fact, we think, it had been among our dental writers, up to that time, but very imperfectly understood. In the text books it was either summarily dismissed with a brief description of its nature, and a still briefer prescription for its cure, viz: extraction; or, if made the subject of more extended remark, it was generally in a theorizing sort of a way, with many speculations in regard to its pathology, and very few practical ideas looking towards its cure.

Our text books are not yet, as a whole, brought up to the present standard of knowledge upon this subject; but it is plain that a great advance has taken place among many members of the profession. Although we had noticed, and read with interest, articles relating to it, in the various dental journals, we were not aware that the subject had been so extensively treated of late, as it really has been, until having committed ourselves to write upon it, we concluded to look back and see what had been already written. In doing this, we discovered that in a file of the issue of 1870 of a single dental journal, were no less than twenty-seven pages devoted to this subject; and in that of the same journal for the previous year, were eighteen pages. Alarmed at this apparent exhaustion of the subject, we ventured not to open the index of any other journal, lest we should be convinced that our occupation was entirely gone. And now, in attempting to present this subject, we do so, as we stated at the outset, with extreme hesitancy, lest we should, apparently, degenerate into a mere copyist, and should fail of advancing a single idea which is not already threadbare. When so much has been said, and so well said, by those abundantly qualified to instruct, it can scarcely be possible to treat the subject without going over substantially the same ground. We shall, therefore, in order to render you any service whatever, confine ourselves strictly to practicalities. No discussion of the origin of pus cells, or their identity with white blood corpuscles; of pyogenic membranes, or microscopical phenomena, will here find place. While we cheerfully recognize the necessity for such investigation and discussions. we think that largest results will, in this instance, be realized by a complete ignoring thereof. We, therefore, proceed at once to a statement of the pathology of the disease in question.

*Pathology.* And here we are again met by the fact before stated, that alveolar abscess is but the sequel to, or advanced stages of alveolar periodontitis. In ordinary cases it does not exist without having been preceded, at some time, by acute symptoms of that disease. We are obliged, however, to state that there are certain exceptions to this rule; at least it is not unheard of, to find teeth with "ulceration" at their roots, without ever having given rise to any acute symptoms, so far as the patient can inform us. The pulp has, in these cases, become devitalized, and all the steps necessary to the formation of an abscess, have been accomplished in such a gradual manner, there seems but the result to tell the tale. But, admitting that as a rule, periodontitis is the necessary forerunner of alveolar abscess, we must conclude that any of the causes producing that disease, may if the irritation be sufficient, give rise to alveolar abscess. That is, applying the general principles of inflammation and its results to this special case, if periodontitis, or acute inflammation of the investing membrane of the root of the tooth, be sufficiently intense in character to devitalize any particle of matter, then pus is formed, and a collection of pus is called and abscess; being without the socket or alveolus, it is called an alveolar abscess; but it is in all respects similar to any other collection of pus of equal amount, when we consider the character of the tissues within which it is formed. A common boil, though simply a collection of pus in the soft tissues, often gives rise to excruciating suffering; how easily, then, can we understand that when the abscess is confined within unyielding walls of bone, and in close proximity to nerves of acute sensibility, the results should be generally exceedingly grave.

Pus, if formed in any quantity, must find an exit. In order to do this, in this case, the bony walls must be pierced by the action of the absorbents, or some other channel of communication with the external surface must be established. This is usually effected by the most direct route; in the incisors and cuspids, the external alveolar plate being thin, is usually punctured; but at other times the pus burrows along the hard palate and discharges behind the soft palate; or penetrates the antrum of Highmore and thence through the nares; or again through the substance of the cheek. In the lower jaw it is usual to find the discharge nearly opposite the root of the diseased tooth, but it is not uncommon to find it making its exit through an opening at the base of the jaw, or at the angle.

*Causes.*—The causes which may give rise to this disease are really somewhat numerous, having been enumerated in treating of periodontitis. But, practically, it will be found that those causes which, after the disease is established, are within our reach, and which we are consequently obliged to take into account in the therapeutical treatment, are confined to two, viz:

1st. Putrescent pulp tissue; and,

2d. Necrosis. This last may be subdivided into two divisions; necrosis of a tooth or root, and necrosis of portions of the alveolus and maxillæ.

An additional cause which, next to these two, is perhaps, the most unfrequent one, is undue irritation of the tooth from biting threads or hard substances; but as we cannot remove this cause, we have nothing to do with it, in the treatment.

*Symptoms.*—These, up to a certain point, will of course be identical with those of periodontitis. Extreme sensitiveness of the tooth to pressure or tapping; intense pain, and extensive swelling, are the well known phenomena which mark the establishment of alveolar abscess. When pus is formed, there is usually some diminution of intense pain, but a constant and generally increasing dull pain until the pus is evacuated. The swelling is sometimes so extensive as to completely metamorphose the patient, running up the alæ of the nose, or along the submaxillary glands and even down the neck, if in the inferior maxilla. The upper lip is often protruded, and the eye almost closed. The soft tissues of the roof of the mouth are sometimes distended to the size of a walnut, and the whole buccal cavity is in a high state of inflammation.

If the disease comes from the first mentioned cause, the *color* of the tooth will form an important diagnostic sign. A dark bluish appearance will generally indicate the presence of a dead pulp within the tooth, through the departure from the normal color may be so slight as to escape observation by any but a practiced eye. But a reflection of light to the palatal surface of the tooth, by a mouth mirror, will usually disclose an absence of that *lively* or life-like appearance which characterizes a healthy tooth. The tooth is generally more or less loosened in its socket; and by these two symptoms it is often possible in an instant to detect the seat of trouble. In chronic cases, however, it is sometimes difficult to locate the cause of the abscess. In such a case the teeth must be carefully examined, and all suspicious fillings closely scrutinized, the history of the teeth being ascertained by inquiry: Had they ached before filling? Were there throbbing, shooting, pains? Did a season of quiet intervene between that trouble and the symptoms which preceded the formation of abscess? If these questions are answered affirmatively, and if the suspected tooth does not respond to thermal tests, we may reasonably suppose that the cause of the abscess is within that tooth. By suspicious fillings, we do not mean poor gold fillings; on the contrary, those well inserted, and apparently "all right," are most likely to be found in connection with a dead pulp. The conducting properties of such a filling have probably caused inflammation and death of the pulp; or perhaps arsenic has been used to obtund sensibility, and has not been confined to that result in its action.

When necrosis of a small portion of the tooth or root is the cause, it cannot be diagnosed from the first mentioned one, by any tests which are readily applied. When the whole tooth—or root— is necrosed, however, such a condition will be easily detected by the color of the tooth, the cleavage of the gums, and other symptoms. But when portions of the alveolar plates or of the maxillæ are necrosed, the probe will reveal the fact.

*Treatment.*—This will, of course, be in a measure dependant upon the cause. Having, by the signs just mentioned, ascertained pretty certainly, the existance of a dead pulp within the tooth, the first step in the treatment must be its complete removal. In order to do this an entrance must be effected into the pulp cavity in such a manner as will enable us thorougly to accomplish our object. If the tooth is highly inflamed, it will not be possible for us to do more than make an opening through which the pus may find vent, at the first sitting. If we are able to establish a communication through the root, we should make it as free as practicable—taking care not to force any debris through the foramen, nor to close the canal with it. If the opening is satisfactorily accomplished, the patient may be dismissed with the assurance that the pain will subside speedily, probably from that moment. A piece of cotton may be loosely placed in the tooth to prevent food, etc., from becoming impacted in it. In a day or two, or as soon as the inflammation has departed, a more thorough opening should be made, the pulp cavity enlarged with suitable instruments, and the root canals also enlarged and cleansed. Wipe them out with cotton dipped in carbolic acid or creosote, and endeavor to saturate the whole cavity with one of these substances. In all these and subsequent manipulations, care must be taken not to stop the canals or break off instruments in them.

But we may fail to afford relief by opening into the tooth. We may be unable to establish a communication through the canal; or the root may be crooked at the point, so that the drill instead of following the canal, will come out at the side of the root. In this case the treatment will not reach the seat of the disease, and the flexible drills sold at the depots will be found of little use, and very liable to break off. We have now the choice of cutting or drilling through the alveolar plate, to give exit to the pus; of extracting the tooth; or of hastening on the suppuration by stimulants. As soon as the location of the abscess manifests itself, the lancet should be freely used, which will at once afford relief.

We have heretofore been considering cases in which an abscess has has been formed at the time of our first seeing the patient, but its contents have not been evacuated. We have seen that if an attempt to save the tooth is to be made this evacuation must be accomplished in one or two ways —either through the tooth, or alveolus. But suppose at our first inspection, we find that a fistulous opening has already been established. The acute inflammation will then have passed away, and the abscess is now in a chronic stage. Pus is either constantly discharged, or at intervals, when the patient "takes cold in the tooth," as he expresses it, a greater or less degree of soreness is noticeable; the gum swells somewhat, but the bony walls not being restored, the soft tissues soon yield, and the discharge is re-established. In these cases there is not sufficient irritation to cause a constant discharge, and thus keep the fistula open. Or, it may be, that there is a constant oozing of pus through the tooth itself, or through some concealed fistula, so slight as to escape our notice. Before we attempt any medication in these cases, we must, as before, enter and cleanse the pulp cavity. There being no inflammation in the pulp tissues, this will be easily accomplished. Having done this as thoroughly as possible, we are ready to begin to adopt the medication necessary to restore the parts to health.

*Medication.*—As we have previously stated, it was formerly considered that a tooth giving rise to a discharge of pus could not be cured. But in the present more advanced stage of dental knowledge, we can, without hesitation, affirm that alveolar abscess is in eight or nine cases out of ten, a curable disease. In fact, when arising from the cause now under consideration, we are disposed to regard it as nearly amenable to treatment, when circumstances are favorable, as an exposed pulp.

The two agents most to be relied on in securing the result, are *patience* and *iodine.* Carbolic acid also, is a necessary adjunct. The patient must be prepared, if necessary to submit to an extended, though by no means painful treat ment, and the practitioner must remember that the secret of his success will be *thoroughness.* We have seen that the cause of the disease is the presence within the teeth of decomposing material generating gas. Upon the complete removal and neutralization of this matter, the discharge would entirely cease, were it not that the parts have assumed an abnormal or diseased condition. The periosteum has become separated from the surface of the root to a greater or less extent; has become thickened and distended, and forms "sac" which contains the pus. In many cases, the complete cleansing of the roots, would alone effect a cure; in other, perhaps a majority, therapeutic agents must be relied on to break up and cause to be absorbed these diseased tissues. In others still, the separation of the periosteum has been so long continued or so extensive that, in consequence of lack of nutrition, necrosis has taken place in the cementum. When this is the case, to any extent, no amount of treatment short of an excision of the necrosed portion, will be likely to effect a cure; since the necrosed portion cannot be exfoliated as with true bone, but unless absorbed or excised must remain to be continually productive of irritation.

The object of our treatment then, will be to break up the diseased structures, and overcome the "habit of disease." The agent to be relied on, as we before observed, is iodine. We use it in combination with creosote or carbolic acid, both because it is thus conveniently applied, and because of the stimulating and antisepic properties which this article possesses. No formula is necessary for this combination. In to a sufficient quantity of carbolic acid, we put iodine enough to saturate it, and leave an excess at the bottom undissolved. As the mixture becomes thick, more acid may be added, or more iodine, when all is dissolved.

We must now endeavor to bring the mixture into contact with the diseased parts. This may be done in either or both of two ways—through the root or roots, and through the fistula, if such exists. To accomplish the former, we wrap a little cotton on a broach, and after dipping it into the mixture, force it up and down in the root. This is, in fact, a sort of a pump, the broach and cotton acting as the piston, and the tincture is thus forced through the foramen and into all the parts. In the majority of cases, the patient will be sensible of the presence of the medicine by a "warm," "burning," or "drawing," sensation, as it is variously described, at the root of the tooth. Great care must be taken not to force the instrument through, or break it off in the root. It is desirable that the patient should be sensible of the presence of the medicine. Should there be a fistulous opening, bubbles of air may, in favorable cases, be seen coming from it, followed, in many instances by the medicine itself. When this occurs, we regard a cure as almost certain, unless necrosis has taken place. It may be necessary to repeat this process many times. We have known it to succeed in apparently very unpromising cases, after one or two applications. On the contrary, in the most obstinate case we ever knew, it was necessary to treat probably thirty times, and for a period of some months. This case, however, resulted in a complete, and so far as we know, permanent cure.

After thoroughly medicating, we prepare to stop the tooth. If there is no fistulous opening, great caution must be observed in doing this. It is not likely that the discharge, if there is one, will be stopped instantly; and should its exit through the tooth be cut off, swelling of the face must be the inevitable result. The root should be filled with a long tent of cotton saturated with the iodine mixture, the end of which is *left protruding*. The external cavity is now filled with cotton dipped in sandarach varnish, and the protruding cotton pushed in, which will unite the whole into one piece; which, should the teeth give trouble, may be withdrawn by the patient. The latter is directed to present himself at once if any soreness supervenes, unless circumstances render it impossible, in which case, if the tooth becomes really troublesome, he should withdraw the cotton. We may expect some throbbing, possibly soreness, and some swelling of the gum. If no particular uneasiness is felt, however, the cotton should not be disturbed, but if the throbbing increases it should be at once withdrawn, which will give relief. If there is a fistulous opening, we have no hesitation in stopping the tooth tightly, as any discharge of pus will find exit by this means, and the patient will not be obliged to withdraw the cotton. But where none exists, we may be unable at first to do more than stop the tooth in the loosest manner, and as the disease is subdued the filling can be more tightly introduced.

Before dismissing our patient, the fistulous opening, if there is one, may be attended to. We are in the habit of introducing a tent of cotton dipped in the iodine solution, as nearly to the apex of the root as possible, by means of a small probe. Care should be taken not to have an excess of the medicine on the cotton, as it will find its way where it is not wanted, upon the lips and gums. The end of the cotton should be cut off short enough to be pushed into the fistula. The object of this cotton is to keep the external part from healing till the secretion of pus has stopped, and the internal parts are healed. If the treatment has been successful, very little more pus will be secreted, and the parts will at once begin to heal by granulations. The cotton will, in the course of two or three days, work out of the orifice, which will t en close up. Its exit, however, should be rather retarded than accelerated, and the end should be cut off as it protrudes, that it may not be removed until it comes entirely out.

But, on the contrary, if the treatment has not effected a cure, the cotton will remain unmoved, and should be withdrawn at the first opportunity.

We allow everything to take its own couree for a week or two. If at the end of that time, it would seem that no success has attended our treatment, we have only two words to say—repeat it. Success is attainable by these means, where the only cause is the presence of a dead pulp, which is the case in a majority of instances, that is, if the tooth is so situated that complete cleansing is possible, and the medicine can be brought into contact with the diseased tissues. When, however, the discharge of pus has been continued so long that necrosis of a portion of the root has occurred, it will, so long as the necrosed portion remains, be impossible to effect a cure And this leads us to a consideration of our second cause, viz: necresis.

This, it will be recollected, we subdivided into two parts, necrosis of the tooth or root, and necrosis of portions of the alveolus and maxillæ. If a necrosed root is present, it must of course, be extracted, and usually this will be advisable, if any portion of a tooth is thus diseased. The only alternative, in order to save the tooth, is to excise the necrosed portion, in which case the remainder of the tooth may assume a healthy condition and be tolerated by the surrounding structures. This may be accomplished either by cutting through the alveolus with a suitable instrument, and cutting and scraping away the necrosed portion; or the tooth may be extracted, the necrosed portion excised, the end polished and the tooth replaced. Though it would seem almost impossible that that this operation should be successful, yet the well known fact that a tooth may be successfully replaced after being removed, and even after being out of the socket for a considerable length of time, would lead us to believe that the cases of this practice which have been reported, are not imaginary ones. But we should regard the attempt to save the tooth, when necrosis has taken place, in the light of an experiment, and should not be surprised if it did not succeed.

When portions of the alveolus and maxillæ are necrosed, they must be removed, either by the processes of nature, or by an operation, when, with the application of an astringent and detergent wash, the discharge will cease.

A few words relative to cases which either have already discharged from the outside of the face, or give evidence of speedily doing so. This result it is very important to prevent if possible, as the resulting scar is a serious disfiguration, particularly in the case of a female. It is generally produced by poultices, or by lancing by ignorant dental or medical practitioners. In case the skin is not already broken, no effort should be spared to prevent the external discharge. We should consider first the advisability of attempting to save the tooth. If in the upper jaw, and valuable, the attempt may be made, but in the lower jaw, gravitation and tension are against us, and this is an argument for extraction. If we decide to retain the tooth, the pulp cavity should be cleansed; then the lancet should be freely used from the *inside.* Evacuate the pus, if possible. Apply sedative and astringent applications externally, tannin, vinegar, laudanum, or arnica and water. Cold applications may also be found useful. It may be advisable to use a compress and bandage, to prevent the external breaking. No dentist should ever put a lancet into the cheek under any circumstances.

In case the external discharge has already taken place, we should consider, first, the sex of the patient, and second, the situation of the fistula; our object should be to heal the orifice without the disfiguration of a scar. If the patient is a male, the scar will probably be hidden by the whiskers, or it may in either sex, be so located as to be out of sight beneath the chin, in either of which cases the tooth may at once be extracted. Should it be found difficult to decide upon the tooth giving rise to the discharge a silver or platinum wire may be passed along the fistula till it strikes against the root of the tooth. But if the opening is in sight especially in the case of a female, the tooth should not be at once extracted, as great depression of tissue will result in cicatrization. An artificial fistula should be made from the inside with the lancet, and the original opening healed by granulation, after which the tooth may be extracted.

